# Phase I/II study of adding intraperitoneal paclitaxel in patients with pancreatic cancer and peritoneal metastasis

**DOI:** 10.1002/bjs.11792

**Published:** 2020-07-07

**Authors:** S. Yamada, T. Fujii, T. Yamamoto, H. Takami, I. Yoshioka, S. Yamaki, F. Sonohara, K. Shibuya, F. Motoi, S. Hirano, Y. Murakami, H. Inoue, M. Hayashi, K. Murotani, J. Kitayama, H. Ishikawa, Y. Kodera, M. Sekimoto, S. Satoi

**Affiliations:** ^1^ Gastroenterological Surgery Nagoya University Graduate School of Medicine Nagoya Japan; ^2^ Department of Surgery and Science Faculty of Medicine, Academic Assembly University of Toyama Toyama Japan; ^3^ Department of Surgery Kansai Medical University Hirakata Japan; ^4^ Department of Surgery Tohoku University Graduate School of Medicine Sendai Japan; ^5^ Department of Surgery, Institute of Biomedical and Health Sciences Hiroshima University Hiroshima Japan; ^6^ Department of Gastroenterological Surgery II, Faculty of Medicine Hokkaido University Sapporo Japan; ^7^ Department of Hepatobiliary–pancreatic and Breast Surgery Ehime University Graduate School of Medicine, Ehime Fukuoka Japan; ^8^ Biostatistics Centre, Graduate School of Medicine Kurume University Fukuoka Japan; ^9^ Department of Gastrointestinal Surgery Jichi Medical University Tochigi Japan; ^10^ Department of Molecular‐Targeting Cancer Prevention, Graduate School of Medical Science Kyoto Prefectural University of Medicine Kyoto Japan

## Abstract

**Background:**

Intraperitoneal chemotherapy using paclitaxel is considered an experimental approach for treating peritoneal carcinomatosis. This study aimed to determine the recommended dose, and to evaluate the clinical efficacy and safety, of the combination of intravenous gemcitabine, intravenous nab‐paclitaxel and intraperitoneal paclitaxel in patients with pancreatic cancer and peritoneal metastasis.

**Methods:**

The frequencies of dose‐limiting toxicities were evaluated, and the recommended dose was determined in phase I. The primary endpoint of the phase II analysis was overall survival rate at 1 year. Secondary endpoints were antitumour effects, symptom‐relieving effects, safety and overall survival.

**Results:**

The recommended doses of intravenous gemcitabine, intravenous nab‐paclitaxel and intraperitoneal paclitaxel were 800, 75 and 20 mg/m^2^ respectively. Among 46 patients enrolled in phase II, the median time to treatment failure was 6·0 (range 0–22·6) months. The response and disease control rates were 21 of 43 and 41 of 43 respectively. Ascites disappeared in 12 of 30 patients, and cytology became negative in 18 of 46. The median survival time was 14·5 months, and the 1‐year overall survival rate was 61 per cent. Conversion surgery was performed in eight of 46 patients, and those who underwent resection survived significantly longer than those who were not treated surgically (median survival not reached *versus* 12·4 months). Grade 3–4 haematological toxicities developed in 35 of 46 patients, whereas non‐haematological adverse events occurred in seven patients.

**Conclusion:**

Adding intraperitoneal paclitaxel had clinical efficacy with acceptable tolerability.

## Graphical abstract

This multicentre phase I/II study was undertaken to determine the recommended dose of the combination of intravenous gemcitabine, intravenous nab‐paclitaxel and intraperitoneal paclitaxel in patients with pancreatic ductal adenocarcinoma and peritoneal metastasis, and to evaluate its clinical efficacy and safety. This regimen exhibited promising clinical efficacy, with acceptable tolerability.

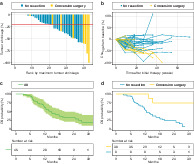

Effective and safe in pancreatic cancer

## Abstract (es)

**Antecedentes:**

La quimioterapia intraperitoneal con paclitaxel se considera una terapia experimental para el tratamiento de la carcinomatosis peritoneal. Este estudio tuvo como objetivo determinar la dosis recomendada y evaluar la eficacia clínica y la seguridad de la combinación de gemcitabina intravenosa, nab‐paclitaxel intravenoso y paclitaxel intraperitoneal en pacientes con cáncer de páncreas y metástasis peritoneales.

**Métodos:**

Se evaluaron las frecuencias de las toxicidades limitantes de la dosis, y la dosis recomendada se determinó en la fase I. El objetivo principal de la fase II fue la tasa de supervivencia global a 1 año. Los objetivos secundarios fueron los efectos antitumorales, los efectos de alivio de los síntomas, la seguridad y la supervivencia global.

**Resultados:**

Las dosis recomendadas de gemcitabina intravenosa, nab‐paclitaxel intravenoso y paclitaxel intraperitoneal fueron de 800, 75 y 20 mg/m^2^, respectivamente. De los 46 pacientes incluidos en la fase II del estudio, la mediana de tiempo hasta el fracaso del tratamiento fue de 6,0 meses (rango, 0‐22,6). Las tasas de respuesta y de control de la enfermedad fueron del 45% y 95%, respectivamente. La ascitis desapareció en el 40% de los pacientes, y la citología se negativizó en el 39% de los pacientes. La mediana del tiempo de supervivencia fue de 14,5 meses y la tasa de supervivencia global a 1 año del 60,9%. La cirugía de rescate se realizó en ocho (17%) pacientes, y los que se sometieron a cirugía sobrevivieron significativamente más tiempo que los que no fueron tratados quirúrgicamente (mediana de supervivencia no alcanzada *versus* 12,4 meses). Las toxicidades hematológicas de grado 3/4 ocurrieron en el 76% de los pacientes, mientras que los eventos adversos no hematológicos se presentaron en el 15% de los pacientes.

**Conclusión:**

Agregar paclitaxel intraperitoneal tuvo eficacia clínica con una tolerabilidad aceptable. (UMIN000018878)

## Introduction

Pancreatic cancer has a poor prognosis, particularly for disseminated disease^1^. The presence of peritoneal metastasis is often associated with ascites and intestinal obstruction, leading to malnutrition and poor performance status, which could deprive patients of the opportunity to receive chemotherapy^2^. Intraperitoneal chemotherapy appears advantageous owing to higher drug concentrations achieved in the peritoneal cavity, compared with systemic chemotherapy^3^, ^4^, ^5^, ^6^.

Favourable clinical effects of intraperitoneal paclitaxel have been reported in clinical trials of patients with peritoneal metastasis, including those with ovarian^3^, ^4^, gastric^5^, ^6^ and pancreatic^7^ cancer. A previous phase II study^8^ of intravenous and intraperitoneal paclitaxel combined with S‐1 for patients with pancreatic cancer and peritoneal metastasis demonstrated good outcomes, with favourable response and disease control rates. The median survival time and 1‐year overall survival rate were 16·3 months and 62 per cent, and conversion surgery was performed in one‐quarter of the enrolled patients^8^. Recently, nab‐paclitaxel combined with gemcitabine was shown to be the standard treatment option for patients with pancreatic cancer and distant metastasis^9^.

The aims of this phase I/II study were to determine the recommended dose for the combination of intravenous nab‐paclitaxel with gemcitabine and intraperitoneal paclitaxel in patients with pancreatic cancer and peritoneal metastasis, and to evaluate its clinical efficacy and safety.

## Methods

**Fig. 1:**
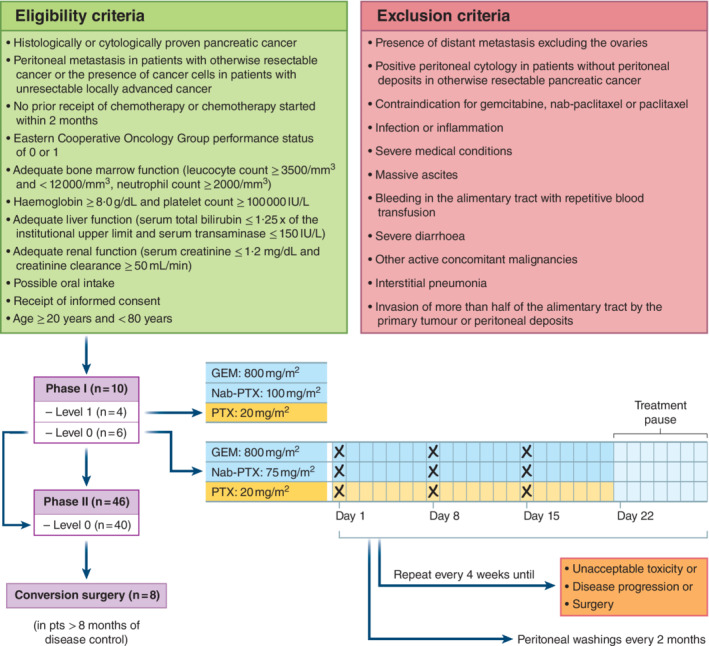
Study protocol and flow chart GEM, gemcitabine; PAX, paclitaxel.

The eligibility and exclusion criteria are shown in *Fig*. *1*. This study was conducted in accordance with the Declaration of Helsinki, and the study protocol was approved by the institutional review board of the affiliated hospital. The registration number for this clinical trial is UMIN000018878. The last follow‐up date was 31 December 2019.

**Table 1:** Clinical responses to treatment

	No. of patients* (*n* = 46)
**Tumour shrinkage (%)** **†**	20 (0–100)
**CA19‐9**	
Minimum (units/ml)†	72 (4–23 700)
Decreased ratio (%)†	84·4 (16·9–99·1)
Normalization	12
**Objective tumour responses**	*n* = 43
Best RECIST category	
Complete response	2
Partial response	19
Stable disease	20
Progressive disease	2
Response	21
Disease control	41
**Peritoneal cytology turned negative**	18
**Disappearance of ascites**	12 of 30
**Conversion surgery**	8

* Unless indicated otherwise;

† values are median (range). CA19‐9, carbohydrate antigen 19‐9; RECIST, Response Evaluation Criteria in Solid Tumours.

### Treatment

If peritoneal dissemination or positive peritoneal cytology was detected during staging laparoscopy or open laparotomy, a peritoneal access port was implanted in the lower abdomen. Intravenous nab‐paclitaxel combined with gemcitabine was administered along with intraperitoneal paclitaxel on days 1, 8 and 15, followed by 1 week of rest. The treatment course was repeated every 4 weeks until unacceptable toxicity had developed, disease progression or surgery (*Fig*. *1*). The criteria for surgical resection (conversion surgery) were: an Eastern Cooperative Oncology Group performance status of 0 or 1; marked tumour shrinkage; decrease or normalization of tumour marker levels; washing cytology via peritoneal access port turned negative (twice in a row); and disappearance of peritoneal deposits on staging laparoscopy^8^. To obtain a sufficient clinical effect with this regimen and avoid early peritoneal recurrence, the decision to proceed to conversion surgery was based on an interval exceeding 8 months between the initial treatment and surgical resection, which was associated with favourable prognosis in patients with initially unresectable pancreatic cancer in a previous study^10^.

### Primary and secondary endpoints

The primary endpoint of phase II of the study was the 1‐year overall survival rate. The secondary endpoints were antitumour effects, symptom‐relieving effects, safety and overall survival.

Objective tumour responses were classified according to Response Evaluation Criteria in Solid Tumours (RECIST) guidelines version 1.1^11^. To evaluate antitumour effects on peritoneal metastases, peritoneal washing cytology specimens were examined every 2 months.

Toxicity was monitored weekly and graded according to the National Cancer Institute–Common Terminology Criteria for Adverse Events (CTCAE) version 4.0^12^.

### Definition of dose‐limiting toxicities and determination of recommended dose

The frequencies of dose‐limiting toxicities were evaluated, and the recommended dose was determined during phase I of the study. Dose‐limiting toxicities were determined during the first two cycles of chemotherapy. Dose‐limiting toxicities were defined according to CTCAE version 4.0^12^ based on the presence of one or more of the following events: grade 4 leucopenia or neutropenia; grade 3 neutropenia complicated by fever of at least 38°C; grade 3–4 anaemia, thrombocytopenia or non‐haematological toxicities; and more than 2 weeks of drug withdrawal within one cycle. The maximum tolerated dose was determined, and the previous level was set as the recommended dose^13^.

### Statistical analysis

The sample size was calculated for an estimated overall survival rate at 1 year after treatment initiation for patients with metastatic pancreatic cancer of 25 per cent. Assuming a null hypothesis of 25 per cent and an alternative hypothesis of 45 per cent with a one‐sided type I error of 0·05 and power of 0·8, enrolment of 24 patients was required.

Continuous variables are expressed as median (range). Overall survival was defined as the interval from the start of treatment to death from any cause. Survival analysis was based on the Kaplan–Meier method, with evaluation of differences using the log rank test. A binary logistic regression model using the backward method was employed to predict the use of conversion surgery. The level of statistical significance was set at *P* < 0·050. All statistical analyses were done using JMP® Pro version 14.2.0 (SAS Institute, Cary, North Carolina, USA).

## Results

A total of 50 patients diagnosed with pancreatic cancer and peritoneal metastasis were enrolled in this phase I/II study from seven Japanese centres; ten patients participated in phase I and 46 (including 6 patients from phase I) in phase II (*Fig*. *1*).

### Determination of recommended dose

Dose levels and dose‐limiting toxicities in phase I are shown in *Table S1* (supporting information). At dose level 1, three of four patients experienced dose‐limiting toxicities. Therefore, the next six patients were enrolled at level 0; only one patient experienced a dose‐limiting toxicity (grade 4 neutropenia) at this level. Based on these results, the recommended doses for intravenous gemcitabine, intravenous nab‐paclitaxel and intraperitoneal paclitaxel were 800, 75 and 20 mg/m^2^ respectively.

### Patient characteristics

A total of 46 patients were enrolled in phase II, and drugs were administered at the recommended dose (level 0). The tumour was located in the pancreatic head in 13 patients and the body/tail in 33. Median tumour diameter was 36 (range 18–64) mm. Primary tumours were categorized as resectable in 12 patients, borderline resectable in 11, and unresectable and locally advanced in 23 patients^14^. Malignant ascites was observed in 30 of the 46 patients on laparoscopy or laparotomy. All patients had positive intraperitoneal cytology, and 29 had pathological confirmation of peritoneal dissemination. The median duration of treatment was 6·0 (range 0–22·6) months (*Table S2*, supporting information).

### Clinical responses and survival by treatment type

**Fig. 2:**
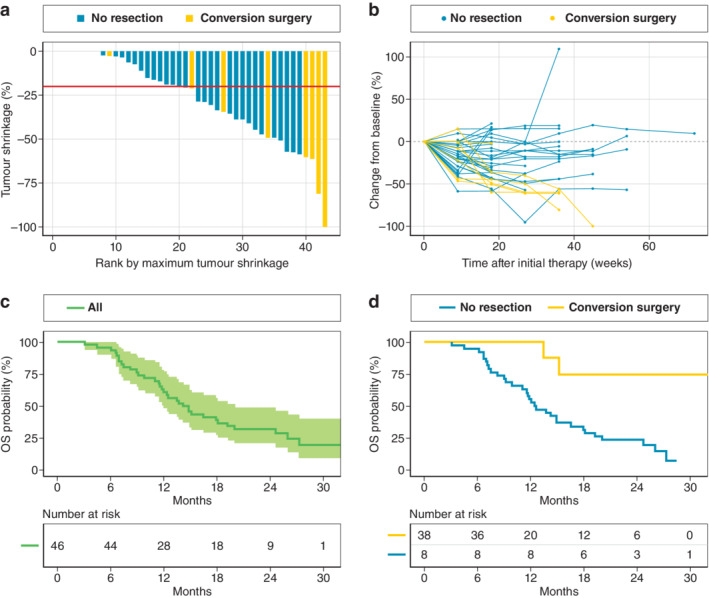
Tumour response and survival **a** Waterfall plot of tumour shrinkage response. Median primary tumour shrinkage was 20 per cent (red line). **b** Spider plot showing tumour response over time. The dotted line indicates no change. **c** Overall survival of 46 patients with pancreatic ductal adenocarcinoma and peritoneal metastasis. The shaded area represents the 95 per cent confidence interval. **d** Comparison of survival between patients who underwent conversion surgery and those who did not. *P* = 0·004 (log rank test). OS, overall survival.

During treatment, median primary tumour shrinkage was 20 (range 0–100) per cent (*Fig*. *2a*,*b*). CA19‐9 levels decreased by a median of 84·4 (range 16·9–99·1) per cent, and normalized in 12 patients. The response and disease control rates were 21 of 43 and 41 of 43 respectively. Peritoneal washing cytology turned negative in 18 of 46 patients, and malignant ascites disappeared in 12 of 30 (*Table* *1*).

**Table 2:** Clinical characteristics of patients who underwent conversion surgery

Patient no.	Age (years)	Sex	Tumour size (mm)*	Best RECIST category	CA19‐9, (units/l)*	Operative procedure	R	Evans grade	Tumour stage	OS (months)	Status
1	74	F	46 → 18	PR	232 → 14	PD + PVR	R0	IIB	T1 N0 M0	13·5	Dead
2	67	F	25 → 10	PR	837 → 48	DP	R0	IIA	T3 N1 M0	32·7	Alive
3	75	M	38 → 25	PR	1127 → 43	DP‐CAR	R0	IIA	T3 N0 M0	15·1	Dead
4	73	F	41 → 40	SD	59 → 47	PD + PVR	R1	IIA	T3 N1 M0	23·4	Alive
5	77	M	30 → 30	SD	246 → 23	DP	R0	IIB	T2 N1 M0	23·4	Alive
6	54	F	25 → 0	CR	167 → 12	DP	R0	IV	T3 N1 M0	17·7	Alive
7	74	M	52 → 10	PR	162 → 37	PD + PVR	R0	III	T3 N0 M0	15·4	Alive
8	77	F	46 → 23	PR	703 → 17	DP	R0	III	T1 N0 M0	14·2	Alive

* Change from before treatment to before surgery. RECIST, Response Evaluation Criteria in Solid Tumours; CA19‐9, carbohydrate antigen 19‐9; OS, overall survival; PR, partial response; PD, pancreatoduodenectomy; PVR, portal vein resection; DP, distal pancreatectomy; DP‐CAR, distal pancreatectomy with coeliac artery resection; SD, stable disease; CR, complete response.

All eligible patients were followed up for at least 12 months. Median overall survival was 14·5 (range 11·5–19·2) months, and 1‐ and 2‐year overall survival rates were 61 and 32 per cent respectively (*Fig*. *2c*).

### Conversion surgery

Eight of the 46 patients underwent conversion surgery (*Table* *2*). The tumour was located in the pancreatic body and tail in seven patients. Six patients had peritoneal dissemination at diagnosis, and two patients had positive peritoneal washing cytology plus unresectable locally advanced cancer before surgery. The median time to surgery was 8·8 (range 4·1–12·2) months after the initiation of chemotherapy. Seven patients underwent R0 resection. The Evans (tumour regression) grade was IIA in three patients, IIB and III in two patients each, and IV in one patient.

Concerning overall survival, patients who underwent conversion surgery survived significantly longer than those who did not (median survival not reached *versus* 12·4 (range 11·0–18·1) months; *P* = 0·004) (*Fig*. *2d*).

### Adverse event profile

The adverse events data are summarized in *Table S3* (supporting information). Grade 3–4 haematological adverse events occurred in 35 of 46 patients, including leucocytopenia (22), neutropenia (32), febrile neutropenia (4), anaemia (8) and thrombocytopenia (6). Grade 3–4 non‐haematological adverse events occurred in seven patients, including appetite loss (4) and nausea (2). A grade 3–4 peritoneal port problem was observed in one patient.

### Prediction of conversion surgery in patients with peritoneal dissemination

Univariable analysis identified a shift to negative peritoneal cytology and normalization of CA19‐9 levels as significant predictors of survival. In multivariable analysis, age (odds ratio 1·29, 95 per cent c.i. 1·04 to 1·59; *P* = 0·020) and a shift to negative peritoneal cytology (odds ratio 32·73, 2·71 to 395·30; *P* = 0·006) were significant predictors of eligibility for conversion surgery (*Table S4*, supporting information).

## Discussion

This trial demonstrated the clinical efficacy of a chemotherapy regimen comprising intravenous gemcitabine, intravenous nab‐paclitaxel and intraperitoneal paclitaxel, with acceptable tolerability, in patients with peritoneal metastasis from pancreatic cancer. Although the clinical response and survival data did not exceed those of an S‐1‐based regimen in a previous study^8^, this strategy represents an option for treating peritoneal disease in countries where S‐1 is not available.

Intraperitoneal chemotherapy enables peritoneal deposits to be exposed to high concentrations of drugs without increasing the systemic concentration to toxic levels^15^. The duration of effectiveness after intraperitoneal administration is determined by the molecular characteristics of the drug. In this regard, paclitaxel is a large‐molecule lipophilic drug that is absorbed slowly^3^. In addition to this pharmacokinetic advantage, combination with systemic chemotherapy is a key variable in intraperitoneal chemotherapy. Ishigami and colleagues^16^ established the use of intravenous/intraperitoneal paclitaxel combined with S‐1 therapy in patients with gastric cancer, and conducted the phase III PHOENIX‐GC trial to compare this regimen with standard therapy. The present authors^8^ also reported the promising clinical efficacy and acceptable tolerability of intravenous/intraperitoneal paclitaxel combined with S‐1 therapy in patients with pancreatic cancer and peritoneal metastasis. In the present study, intraperitoneal paclitaxel was added to the combination of intravenous gemcitabine and intravenous nab‐paclitaxel, which has been established as a standard therapy for metastatic disease^9^, and its efficacy was confirmed to be similar to that reported previously for intravenous/intraperitoneal paclitaxel and S‐1 therapy.

A previous study^17^ reported poor overall survival following weekly paclitaxel in patients with pancreatic cancer and malignant ascites. Another study^2^ revealed median survival times of 8 months in patients with pancreatic cancer and peritoneal dissemination, and 13 months in those with locally advanced disease and positive peritoneal washing cytology. Considering that patients with peritoneal metastasis generally have a poor prognosis, the results of the present study may be considered encouraging. Conversely, no significant improvement was noted compared with the effects of a previous S‐1‐based regimen^8^, despite the use of state‐of‐the‐art systemic therapy in combination with intraperitoneal paclitaxel.

Recently, multidisciplinary treatment combining chemotherapy and surgery has been used widely and regarded as a promising strategy. In particular, conversion surgery for metastatic disease has an advantage in that chemotherapy is administered to patients with a better performance status. The combination therapy used in the present study enabled eight of 46 patients to be eligible for conversion surgery. The median survival time was not reached in patients who underwent conversion surgery, which is a considerable achievement given the generally poor outcomes of patients with pancreatic cancer and peritoneal disease. Median survival time after conversion surgery for pancreatic cancer has generally been reported in the range 30–52 months^10^, ^18^, ^19^, ^20^. The present combination therapy has performed remarkably in terms of both conversion rate and survival outcome, and its potential to control both peritoneal metastasis and the primary tumour was proven. However, this investigation was conducted as a phase I/II study with a single‐arm design; the bias in its clinical implications must be recognized. A phase III study is being planned to compare survival outcomes between the intraperitoneal therapy used here and standard chemotherapy.

Regarding adverse events, grade 3–4 haematological toxicities occurred in 35 of 46 patients and non‐haematological adverse events in seven. In particular, the rate of haematological toxicities was high, but the incidence and severity were comparable to those of standard chemotherapy regimens^9^, ^21^ and previous findings^8^. In the phase II analysis, grade 4 neutropenia was noted in seven of 40 patients (18 per cent); however, these events were well managed and tolerable. Intraperitoneal port‐related adverse events were less frequent than in the authors' initial experience^8^, which was a meaningful result for this intraperitoneal therapy.

## Supporting information


**Appendix S1:** Supporting information
