# Supplementary material for: Phase I/II study of adding intraperitoneal paclitaxel in patients with pancreatic cancer and peritoneal metastasis
Source: Br J Surg. 2020 Jul 7;107(13):1811–7. doi: 10.1002/bjs.11792 (PMC7689756; doi:10.1002/bjs.11792)
Supplement: Supplementary file 1 — Appendix S1: Supporting information [file BJS-107-1811-s001.docx]

**BJS11792**

**Phase I/II study of adding intraperitoneal paclitaxel in patients with pancreatic cancer and peritoneal metastasis**

S. Yamada, T. Fujii, T. Yamamoto, H. Takami, I. Yoshioka, S. Yamaki, F. Sonohara, K. Shibuya, F. Motoi, S. Hirano, Y. Murakami, H. Inoue, M. Hayashi, K. Murotani, J. Kitayama, H. Ishikawa, Y. Kodera, M. Sekimoto and S. Satoi

| **Table S1 Dose levels of gemcitabine, nab-paclitaxel, and paclitaxel in the phase I portion** | | | | | |
| --- | --- | --- | --- | --- | --- |
| Dose level | Pts | GEM (mg/m^2^) | Nab-PTX (mg/m^2^) | PTX (mg/m^2^) | Dose-limiting toxicity |
| Level −2 | NA | 600 | 50 | 20 | NA |
| Level −1 | NA | 800 | 50 | 20 | NA |
| Level 0 | 6 | 800 | 75 | 20 | Case 1; G4 neutropenia |
| Level 1 | 4 | 800 | 100 | 20 | Case 1; G4 neutropenia/leukocytopenia, G3 febrile neutropenia, G3 thrombocytopenia  Case 2; G5 gastrointestinal hemorrhage  Case 3; G4 neutropenia/leukocytopenia |
| Level 2 | NA | 800 | 125 | 20 | NA |
| Level 3 | NA | 1000 | 125 | 20 | NA |
| Pts, number of patients; NA, not available; GEM, gemcitabine; nab-PTX, nab-paclitaxel; PTX, paclitaxel; G, grade | | | | | |

| **Table S2** **Patient characteristics at diagnosis** | |
| --- | --- |
| Characteristics | Value |
| Age, years | 69 (47–79) |
| Sex, male/female | 26 (56.5)/20 (43.4) |
| BMI, kg/m^2^ | 19.9 (15.1–30.5) |
| Performance status, 0/1 | 35 (76.1)/11 (23.9) |
| Tumor location, head/body and tail | 13 (28.3)/33 (71.7) |
| Tumor size, mm | 36 (18–64) |
| Resectability, R/BR/UR | 12 (26.1)/11 (23.9)/23 (50.0) |
| Ascites, −/+ | 16 (34.8)/30 (65.2) |
| Peritoneal dissemination, 0/1/2–5 /≥6 | 17 (37.0)/1 (2.2)/4 (8.7)/24 (52.2) |
| Peritoneal (washing) cytology, −/+ | 0 (0.0)/46 (100.0) |
| Total protein, g/dL | 6.5 (3.5–7.6) |
| Albumin, g/dL | 3.7 (2.5–4.8) |
| Bilirubin, mg/dL | 0.7 (0.3–1.7) |
| CA19-9 level, U/mL | 653 (3-38,000) |
| CA125 level, U/mL | 49.7 (9-280) |
| DUPAN-2 level, U/mL | 750 (25-96,500) |
| Biliary drainage, −/+ | 36 (78.3)/10 (21.7) |
| Duration of protocol therapy, months | 6.0 (0–22.6) |
| Post-line therapy, FFX/S-1/GnP/others/none | 10 (21.7)/4 (8.7)/2 (4.3)/15 (32.6)/15 (32.6) |
| Data are presented as the median (range) or n (%).  Abbreviations: BMI, body mass index; CA19-9, carbohydrate antigen 19-9; CA125, carbohydrate antigen 125; R, resectable; BR, borderline resectable; UR, unresectable; FFX, FOLFIRINOX; GnP, nab-paclitaxel combined with gemcitabine | |

| **Table S3 Profile of adverse events** | | | | | |
| --- | --- | --- | --- | --- | --- |
| Grading by CTCAE v4.0 | grade 1 | grade 2 | grade 3 | grade 4 | grade 3/4 (%) |
| Hematological | 2 | 8 | 19 | 16 | 76 |
| Leukocytopenia | 2 | 17 | 18 | 4 | 48 |
| Neutropenia | 4 | 5 | 16 | 16 | 70 |
| Febrile neutropenia | 0 | 0 | 4 | 0 | 9 |
| Anemia | 16 | 19 | 8 | 0 | 17 |
| Thrombocytopenia | 23 | 9 | 6 | 0 | 13 |
| Bilirubin | 8 | 1 | 1 | 0 | 2 |
| AST | 23 | 2 | 0 | 0 | 0 |
| ALT | 29 | 1 | 0 | 0 | 0 |
| Albumin | 21 | 14 | 3 | 0 | 7 |
| Creatinine | 7 | 0 | 0 | 0 | 0 |
|  |  |  |  |  |  |
| Non-hematologic | 7 | 32 | 7 | 0 | 15 |
| Fever | 8 | 8 | 1 | 0 | 2 |
| Fatigue | 21 | 14 | 0 | 0 | 0 |
| Appetite loss | 22 | 10 | 4 | 0 | 9 |
| Nausea | 12 | 6 | 2 | 0 | 4 |
| Vomiting | 5 | 4 | 1 | 0 | 2 |
| Diarrhea | 10 | 4 | 1 | 0 | 2 |
| Abdominal pain | 18 | 8 | 1 | 0 | 2 |
| Skin rash | 8 | 0 | 0 | 0 | 0 |
| Oral mucositis | 1 | 1 | 0 | 0 | 0 |
| Peripheral neuropathy | 22 | 5 | 1 | 0 | 2 |
| Alopecia | 9 | 27 | 0 | 0 | 0 |
| Edema | 5 | 3 | 0 | 0 | 0 |
| Pneumonia | 0 | 0 | 0 | 0 | 0 |
| Dysgeusia | 11 | 4 | 0 | 0 | 0 |
| Peritoneal port trouble | 7 | 6 | 1 | 0 | 2 |
| CTCAE, Common Terminology Criteria for Adverse Events; AST, aspartate aminotransferase; ALT, alanine aminotransferase | | | | | |

| **Table S4 Binary logistic regression model to predict the use of conversion surgery in patients with peritoneal dissemination** | | | | | | |
| --- | --- | --- | --- | --- | --- | --- |
| Variables | Univariate | | | Multivariate | | |
|  | OR | 95% CI | P | OR | 95% CI | P |
| Age | 1.12 | 0.975–1.276 | 0.1101 | 1.288 | 1.041–1.593 | 0.0198* |
| Sex (female) | 2.56 | 0.530–12.31 | 0.2421 |  |  |  |
| Performance status (0) | 2.50 | 0.273–22.93 | 0.4178 |  |  |  |
| Tumor location (body and tail) | 3.23 | 0.356–29.27 | 0.2971 |  |  |  |
| Tumor size | 1.02 | 0.954–1.084 | 0.6059 |  |  |  |
| Resectability (resectable) | 1.93 | 0.385–9.720 | 0.4237 |  |  |  |
| CY turned negative | 6.50 | 1.140–37.05 | 0.0350* | 32.73 | 2.709–395.3 | 0.0061* |
| Peritoneal dissemination (positive) | 1.96 | 0.348–11.01 | 0.4464 |  |  |  |
| Ascites (negative) | 2.17 | 0.462–10.16 | 0.3268 |  |  |  |
| Ascites disappearance | 1.60 | 0.193–13.24 | 0.6628 |  |  |  |
| CA19-9 | 1.00 | 0.997–1.000 | 0.1097 |  |  |  |
| CA19-9 decrease ratio | 1.02 | 0.981–1.055 | 0.3663 |  |  |  |
| CA19-9 normalization | 7.38 | 1.418–38.42 | 0.0176* |  |  |  |
| RECIST best (CR and PR) | 4.00 | 0.706–22.67 | 0.1173 |  |  |  |
| Abbreviations: OR, odds ratio; CI, confidence interval; CY, peritoneal cytology; CA19-9, carbohydrate antigen 19-9; RECIST, Response Evaluation Criteria in Solid Tumors; CR, complete response; PR, partial response | | | | | | |
